# Renal Vascular Affection in Systemic Sclerosis Patients and Possible Correlation with Peripheral Vascular Involvement Assessed by Nailfold Capillaroscopy

**DOI:** 10.31138/mjr.290425.vad

**Published:** 2025-12-31

**Authors:** Nermeen Samy, Dalia Fayez, Salma Hassan Tantawy, Rahma A. Elziaty, Nada Mahmoud Abd El-Monem, Amr Mohammad Mohammad Hawwash

**Affiliations:** 1 Rheumatology Unit, Internal Medicine Department, Ain Shams University Medical School, Cairo, Egypt;; 2 Department of Diagnostic and Interventional Radiology and Molecular Imaging, Faculty of Medicine, Ain Shams University, Cairo, Egypt

**Keywords:** proteinuria, creatinine, glomerular filtration rate, microscopic angioscopy, scleroderma, systemic, ultrasonography, doppler, systemic sclerosis, renal duplex, RRI, NFC, DSS

## Abstract

**Background:**

Symptomatic renal involvement was observed in only 24% of systemic sclerosis (SSc) patients.

**Objective:**

We evaluated renal vascular affection, which is linked to disease characteristics and nailfold capillaroscopy (NFC) outcomes in SSc patients

**Methods:**

Fifty SSc individuals were subjected to renal doppler ultrasound, NFC, and evaluation of disease severity utilising the Medsger disease severity scale.

**Results:**

The cohort comprised 46 females and 4 males, with a mean age of 33.82 ± 11.19 years. Symptomatic renal affection [proteinuria, elevated creatinine level, and mitigated creatinine clearance, and estimated glomerular filtration rate (eGFR) ± hypertension] was observed in 24% of subjects. However, an elevated renal resistive index (RRI) was detected in 44%. Accordingly, patients were allocated into Group 1 (patients exhibiting normal RRI, n = 28) and Group 2 (patients manifesting raised RRI, n = 22). Group 2 patients experienced significantly older age, prolonged disease duration, raised severity scores, protein/creatinine ratio, and avascular NFC scores (p < 0.05). The RRI demonstrated a positive association with age, disease duration, severity, duration of Raynaud’s phenomenon, and avascular scores (p < 0.05), alongside negative correlations with creatinine clearance, eGFR, and capillary density (p < 0.05). The main predictors of high RRI encompassed age > 43 years, disease severity score > 5, protein/creatinine ratio > 0.21, capillary density ≤ 6, and avascular score > 1 (p = 0.05).

**Conclusion:**

The RRI detected early asymptomatic renal affection, which correlated positively with age, disease duration, severity, and high NFC avascular scores and negatively with creatinine clearance and eGFR.

## INTRODUCTION

Renal involvement is common in various autoimmune diseases, with its prevalence and severity depending on the underlying condition, ranging from asymptomatic involvement to renal failure.^1^ The hallmarks of systemic sclerosis (SSc), a multifaceted disorder, include immune system malfunction, early microvascular abnormalities, and chronic inflammation, resulting in progressive fibrosis of the skin and internal organs.^2,3^

The range of renal symptoms in SSc ranges from isolated decreases in glomerular filtration rate to heightened intrarenal arterial stiffness, isolated proteinuria, hypertension, renal crisis, and less common manifestations encompassing anti-neutrophilic cytoplasmic antibody (ANCA)-related vasculitis or -phospholipid antibody nephropathy.^4,5^

In SSc patients, renal vascular resistance is elevated in multiple vascular regions, including the renal, inter-lobar, and cortical arteries.^6^ Clinically, renal vasculopathy is linked to the severity of renal involvement and disease activity, as demonstrated by the occurrence of fresh digital gangrene and Raynaud’s phenomenon severity.^7^ Early administration of prostacyclin analogs and angiotensin-converting enzyme (ACE) inhibitors can modulate several outcomes, including modification of renal vascular resistance indices, enhancement of renal blood flow, slow the renal impairment progression, and potentially reverse severe outcomes, including scleroderma renal crisis (SRC).^8^

Nailfold capillaroscopy (NFC) is a non-invasive, efficient, and unique technology that enables rapid performance and is the sole approach for the morphological assessment of nailfold capillaries.^9^ The early emergence of certain microvascular abnormalities that precede diverse pathological occurrences, underpins the predominant focus on capillaroscopy in SSc.^10^ According to Rosato et al. and Bruni et al., renal Doppler indices of intrarenal arterial stiffness are elevated during the progression of capillaroscopic injury, while the observed glomerular filtration rate (GFR) decreases significantly.^6,11^ Here, we aimed to assess renal vascular involvement in SSc patients linked to disease severity and peripheral vascular contribution, as determined by NFC.

## PATIENTS AND METHODS

Typically, 50 SSc patients (age ranged from 18–75 years old), diagnosed based on the ACR/EULAR criteria,^12^ were enrolled in this cross-sectional study. Patients who were current or former smokers, pregnant, had other associated autoimmune diseases, had a history of diabetes mellitus or hypertension prior to disease onset, or had chronic renal disease due to other aetiologies, were excluded. After obtaining informed written consent, patients were enrolled in the inpatient rheumatology department and outpatient clinic of Ain Shams University Hospitals. This study was approved by the Research Ethical Committee of Ain Shams University, following the principles of the Helsinki Declaration (5/12/2023, No. FWA00017585).

A comprehensive medical history and clinical examination were conducted, particularly on SSc clinical data, disease characteristics, and blood pressure evaluation. Arterial hypertension was diagnosed based on the ESH/ESC guidelines, characterised by SBP > 140 mmHg and/or DBP > 90 mmHg.^13^ The limited (LCSS) and diffuse (DCSS) forms were classified employing the modified Rodnan skin score (mRSS) for skin involvement.^14^

The Medsger disease severity scale (DSS) was utilised to assess the disease severity.^15^ The scale evaluates nine organs, assigning scores from 0 (no recorded involvement) to 4 (end-stage disease). Mild, moderate, and severe involvement were categorised as scores of 1, 2, and 3, respectively.

Laboratory investigations included CBC, ESR, lipid profile, liver enzymes (ALT and AST), serum creatinine, blood urea, uric acid, complete urine analysis, protein/creatinine ratio, serological tests (anti-SCL-70 and anti-centromere AB), and estimated GFR deploying the CKD-EPI creatinine 2021 equation^16^ as follows:

eGFR = 142 x min (standardised Scr/K,1), α * max (standardised Scr/K,1), –1.200 * 0.9938, age in years * 1.012 [if female], where Scr represents the serum creatinine concentration (mg/dL), K is 0.7 and 0.9 for females and males, respectively, α is –0.241 and –0.302 for females and males, while min and max (standardised Scr/K,1) denote the minimum and maximum values, respectively.

A renal Doppler ultrasound was conducted using the GE Logiq P9 ultrasound machine. Patients were instructed to lie supine for 15 minutes before the Doppler test. The Doppler probe was positioned at three distinct locations to acquire renal Doppler flow from the interlobar arteries of both kidneys. A single investigator performed the Doppler ultrasonography blinded to the patients’ clinical characteristics.

The subsequent parameters were measured: end-diastolic velocity (EDV), renal artery resistive index (RRI), pulsatile index (PI), peak systolic velocity (PSV), and systolic/diastolic ratio (S/D).^17^ The normal RRI ranges from 0.47 to 0.70, rises with age, and typically manifests a discrepancy of below 5–8% between the two kidneys. The RRI was calculated as $\text{RI}=\frac{\text{PSV}-\text{EDV}}{\text{PSV}}$. Normal PI values range from 0.7 to 1.4, and the renal artery pulsatility index (RAPI) was calculated as RAPI = {(PSV) - (EDV)} / average velocity. For both kidneys, the average of three measurements was calculated for each Doppler parameter for the interlobar arteries.^18^

The same operator performed the NFC using an Optilia video capillaroscope device equipped with a ×500 optical probe. The second, third, fourth, and fifth fingers of both hands were investigated for nailfolds.^19^ The capillaroscopic patterns observed in the “SSc pattern” encompassed early, active, and late phases.^20^ A semiquantitative rating scale was used to score each capillary abnormality identified during NFC for SSc capillaroscopic avascular patterns: 0 (no changes), 1 (< 33% of capillary alterations/reduction), 2 (33–66% of capillary alterations/reduction), and 3 (> 66% of capillary alterations/reduction) per linear millimetre.^21^

Statistical analysis was conducted utilising the Statistical Package for the Social Sciences (SPSS) version 23. Variables were expressed as mean, standard deviation, range, or numbers and percentages. Qualitative data were compared with Fisher’s exact and the chi-square tests. Parametric quantitative data were compared employing the independent t-test, while non-parametric data were analysed utilising the Mann-Whitney test. Spearman correlation coefficients were calculated for correlation analyses. Multivariate and univariate logistic regression analyses were performed to ascertain renal artery resistive index variables. The 95% confidence intervals (CI) and odds ratios were calculated for these factors. A p-value < 0.05 is deemed statistically significant.

## RESULTS

**Figure 1.**
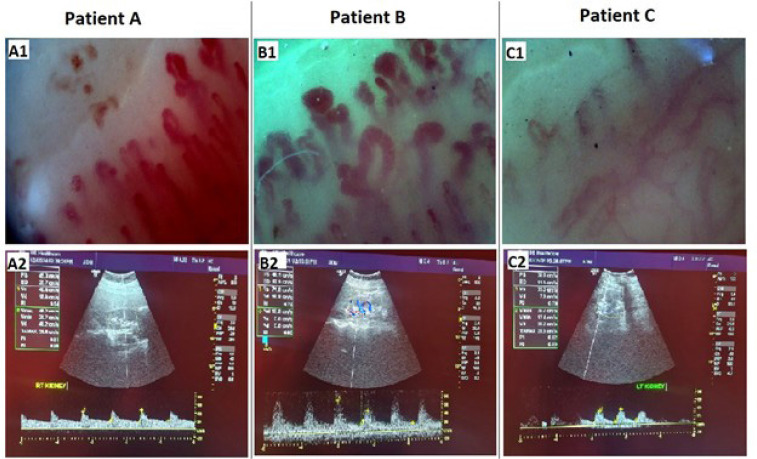
Description of RRI together with NFC results among studied patients. **Patient A. (A1)** NFC shows early scleroderma pattern, normal capillary density (8/mm), presence of giant capillaries and presence of haemorrhage. **(A2)** Renal duplex shows normal RRI=0.54. **Patient B. (B1)** NFC shows active scleroderma pattern, decreased capillary density (5/mm), presence of giant capillaries and presence of haemorrhage. **(B2)** Renal duplex shows high RRI=0.74. **Patient C. (C1)** NFC shows late scleroderma pattern, markedly decreased capillary density (2/mm), presence of neoangiogenesis. **(C2)** Renal duplex shows RRI=0.82 higher than patient B.

Most of the patients were female (92%). The mean age of the cohort was 40.56 ± 10.8 years, spanning from 22 to 73 years. Thirty-three patients were classified as LcSSc, and 17 as diffuse cutaneous systemic sclerosis (dcSSc). Demographic, clinical, and laboratory data, drug history, renal Doppler indices, and NFC results are presented in **Table 1** and **Figure 1**.

**Table 1.** Description of all demographic, clinical, laboratory, drug history together with renal duplex indices and NFC results.

**Variable mean±SD or n(%)**	**SSc patients (n=50)**
**Demographic data**	
Age (years)	40.56 ± 10.8
Sex	
Female	46 (92%)
Male	4 (8%)
Age of disease onset (years)	33.82 ± 11.19
Disease duration (years)	6.73 ± 5.66
Body mass index BMI (kg/m)	27.16 ± 5.24
**Clinical manifestations**	
Extension of skin tightness	
DCSS	17 (34%)
LCSS	33 (66%)
Sclerodactyly	47 (94 %)
Joint pain	
Arthritis	30 (60%)
Arthralgia	18 (36%)
Hand deformities	12 (24%)
Raynaud's	49 (98%)
Duration of Raynaud’s (years)	6.39 ± 6.50
Digital ulcers / scars	35 (70%)
Digital infarction	13 (26%)
Telangiectasia	19 (38%)
Upper GIT symptoms	31 (62%)
GERD	31 (62%)
Dysphagia	28 (56%)
Lower GIT symptoms (constipation, flatulence)	14 (28%)
SBP (mmHg)	109.6 ± 15.48
DBP (mmHg)	69.5 ± 11.75
Hypertension	5 (10%)
Dyspnoea	32 (64%)
Pulmonary hypertension (High RVSP)	6 (12%)
Arrythmias	6 (12%)
Interstitial Lung Disease	28 (56%)
**Medsger disease Severity Score (total score 36) Laboratory investigations**	7.50 ± 4.18
ESR (mm/hour)	40.7 ± 17.75
Triglycerides (mg/dl)	127.34 ± 29.24
Cholesterol (mg/dl)	190.28 ± 25.36
LDL (mg/dl)	117.16 ± 21.19
HDL (mg/dl)	50.92 ± 8.39
Serum creatinine (mg/dl)	0.71 ± 0.247
Urea (mg/dl)	23.5 ± 7.3
Uric acid (mg/dl)	4.02 ± 1.43
Urine analysis	6.18 ± 6.11
Pus cells (HPF)	
Red cells or casts (HPF)	4.52 ± 3.18
Albumin	
Nil	45 (90%)
Trace	1 (2%)
1	3 (6%)
2	1 (2%)
Protein/creatinine ratio (mg/mg creatinine)	0.17 ± 0.12
Normal	47 (84%)
Proteinuria >0.15	3 (6%)
Creatinine clearance (ml/min)	135.4 ± 51.97
Normal (97–137in males, 88–128in females)	38 (76%)
Low	12 (24%)
eGFR (ml/min/1.72m^2^)	108.6 ± 33.64
Normal (>60)	49 (98%)
Low (< 60)	1 (2%)
Anti-scleroderma 70	19 (38%)
Anti-centromere antibodies	22 (44%)
**Renal duplex indices**	
Renal resistive index (RRI)	0.66 ± 0.06
RRI	
Normal	28 (56%)
High	22 (44%)
Pulsatile index (PI)	0.86 ± 0.18
Systolic/diastolic (S/D) ratio	3.07 ± 0.61
**NFC results**	
Capillary density/mm	5.5 ± 1.54
Mean capillary length (micrometre)	235.3 ± 79.47
Mean capillary width (micrometre)	63.94 ± 14.77
Haemorrhage	43 (86%)
Dilated mega capillary	47 (94%)
Tortuous	27 (54%)
Neo-angiogenesis	6 (12%)
Sub-capillary Plexus	14 (28%)
Nailfold capillaroscopy pattern	
Early	14 (28%)
Active	31 (62%)
Late	5 (10%)
Avascular score	1.54 ± 0.84
**Medications Immunosuppression**	
Methotrexate	23 (46%)
Mycophenolate mofetil	20 (40%)
Cyclophosphamide	32 (64%)
Azathioprine	6 (12%)
**Oral steroids**	
Yes	41 (82%)
**Oral steroid Dose (mg)**	
Median (IQR)	10 (5 – 10)
Range	2.5 – 15
**Oral steroid Duration (years)**	
Median (IQR)	2 (1 – 8)
Range	0.08 – 15
**Vasodilators**	
Calcium channel blockers	39 (78%)
PDE-5i (Sildenafil)	19 (38%)
(PGI2 analogue) Ilioprost	5 (10%)
(endothelin receptor antagonist) Bosentan	1 (2%)

BMI: body mass index; SBP: systolic blood pressure; DBP: diastolic blood pressure; LCSS: limited cutaneous systemic sclerosis; DCSS: diffuse cutaneous systemic sclerosis; GERD: gastroesophageal reflux disease; ILD: interstitial lung disease; RVSP: right ventricular systolic pressure; ESR: erythrocyte sedimentation rate; eGFR: estimated glomerular filtration rate; AST: aspartate transaminase; ALT: alanine transaminase; LDL: low density lipoprotein; HDL: high density lipoprotein; HPF: high power field; RRI: renal resistive index; PI: pulsatile index; S/D: systolic/diastolic; NFC: nailfold capillaroscopy.

Symptomatic renal involvement, including proteinuria, elevated creatinine levels, reduced creatinine clearance, and low eGFR ± hypertension, was found in 24% of patients. Elevated RRI was detected in 44% of patients. Based on RRI, a potential biomarker of early renal vasculopathy (even before other parameters appear), patients were allocated into two groups: Group 1 (normal RRI, 28 patients) and Group 2 (high RRI, 22 patients).

Furthermore, group 2 patients exhibited significantly older age, prolonged disease duration, greater dyspnoea, and higher DSS than Group 1 (p < 0.05). Moreover, no significant differences were revealed in drug regimens or laboratory investigations, except for a significantly higher protein/creatinine ratio in Group 2 (p = 0.015).

In terms of NFC findings, Group 2 patients showed significantly reduced capillary density, fewer tortuous capillaries, greater neoangiogenesis, and a greater avascular score (p < 0.05). Active and late capillary patterns were significantly more frequent in Group 2, whereas Group 1 predominantly exhibited the early capillaroscopic pattern (p = 0.003; **Table 2**).

**Table 2.** Comparative data between Group 1 and Group 2.

Variable [mean±SD or n (%)]	Group 1 (normal RRI) No.= 28	Group 2 (high RRI) No.= 22	**Test value**	**P-value**
Age	36.04 ± 9.52	46.32 ± 9.69	–3.762	**<0.0001**
Sex				
Female	26 (92.9%)	20 (90.9%)	0.064*	0.801
Male	2 (7.1%)	2 (9.1%)		
Age of disease onset (years)	30.43 ± 10.69	38.14 ± 10.5	–2.550•	**0.014**
Disease duration (years)	5.59 ± 4.91 0.67 – 20	8.18 ± 6.31 1 – 24	–1.660≠	0.097
Body mass index BMI (kg/m^2^)				
Yes	3 (10.7%)	2 (9.1%)	0.270•	0.789
Range	17.3 – 37.1	18 – 40.8		
Extension of skin tightness				
DCSS	6 (21.4%)	11 (50%)	4.482*	**0.034**
LCSS	22 (78.6%)	11 (50%)		
Sclerodactyly	25 (89.3%)	22 (100.0%)	2.508	0.113
Joint pain				
No	2 (7.1%)	0 (0%)		
Arthritis	13 (46.4%)	17 (77.3%)	5.447*	0.066
Arthralgia	13 (46.4%)	5 (22.7%)		
Hand deformities	4 (14.3%)	8 (36.4%)	3.292*	0.070
Raynaud's phenomenon	27 (96.4%)	22 (100%)	0.802*	0.371
Duration (years)	5.56 ± 5.79	7.70 ± 7.23	–1.455≠	0.146
Digital ulcers	17 (60.7%)	18 (81.8%)	2.613*	0.106
Digital infarction/scar	5 (17.9%)	8 (36.4%)	2.193*	0.139
Telangiectasia	9 (32.1%)	10 (45.5%)	0.927	0.336
Upper GI symptoms	18 (64.3%)	13 (59.1%)	0.141	0.707
Lower GI symptoms	6 (21.4%)	8 (36.4%)	1.363	0.243
SBP (mmHg)	110.36 ± 14.33	108.64 ± 17.13	0.387•	0.701
DBP (mmHg)	71.96 ± 10.74	66.36 ± 12.46	1.706•	0.095
Dyspnoea	13 (46.4%)	19 (86.4%)	8.528*	**0.003**
High RVSP	3 (10.7%)	3 (13.6%)	0.100*	0.752
Arrhythmias	2 (7.1%)	4 (18.2%)	1.422*	0.233
Interstitial Lung Disease	13 (46.4%)	15 (68.2%)	2.366*	0.124
Medsger disease severity score (total score 36)	6.25 ± 3.92	9.09 ± 4.02	–2.593≠	**0.010**
ESR (mm/hour)	41.68 ± 16.37	39.45 ± 19.69	0.436•	0.665
Triglycerides (mg/dl)	121.93 ± 24.85	134.23 ± 33.36	–1.495•	0.142
Cholesterol (mg/dl)	187.46 ± 25.35	193.86 ± 25.5	–0.884•	0.381
LDL (mg/dl)	116 ± 20.05	118.64 ± 22.95	–0.433•	0.667
HDL (mg/dl)	52.14 ± 8.48	49.36 ± 8.21	1.167•	0.249
Serum creatinine (mg/dl)	0.7 ± 0.19	0.72 ± 0.37	–0.372•	0.712
Urea (mg/dl)	22.54 ± 6.21	24.73 ± 8.48	–1.056•	0.296
Uric acid (mg/dl)	3.93 ± 1.57	4.13 ± 1.25	–0.503•	0.617
Urine analysis	6.04 ± 7.41	6.36 ± 4.05		
*Pus cells (HPF)*				
*Red cells or casts (HPF)*	4.61 ± 3.79	4.41 ± 2.26	–0.865≠	0.387
*Albumin*				
Nil	24 (85.7%)	21 (95.5%)	–0.447≠	0.655
Trace	1 (3.6%)	0 (0%)	1.840*	0.606
1	2 (7.1%)	1 (4.5%)		
2	1 (3.6%)	0 (0%)		
Protein/creatinine ratio (mg/mg creatinine)	0.14 ± 0.06	0.22 ± 0.16	2.531	**0.015**
Normal	28 (100%)	19 (86.4%)	5.224	0.073
Proteinuria	0 (0%)	3 (13.6%)		
Creatinine clearance (ml/min)	141.14 ± 46.71	128.09 ± 58.28	0.879•	0.384
Normal	24 (85.7%)	14 (63.6%)		
Low	4 (14.3%)	8 (36.4%)	3.292*	0.070
Estimated GFR (ml/min/1.72m^2^)	115.93 ± 38.57	99.27 ± 23		
Normal	28 (100%)	77 21 (95.5%)	1.776•	0.082
Low	0 (0%)	1 (4.5%)	1.299*	0.254
Serological biomarkers				
*Anti SCL 70 antibody*				
Negative	20 (71.4%)	11 (50.0%)	2.401	0.121
Positive	8 (28.6%)	11 (50.0%)	2.366	0.124
*Anti-centromere antibody*				
Negative	13 (46.4%)	15 (68.2%)		
Positive	15 (53.6%)	7 (31.8%)		
Capillary density/mm	6.14 ± 1.43	4.68 ± 1.29	3.741•	**<0.0001**
Mean capillary length (micrometre)	249.57 ± 72.44	217.14 ± 85.87	1.448•	0.154
Mean capillary width (micrometre)	62.51 ± 13.55	65.77 ± 16.33	–0.773•	0.443
Haemorrhage	25 (89.3%)	18 (81.8%)	0.571*	0.450
Dilated mega capillary	27 (96.4%)	20 (90.9%)	0.665*	0.415
Tortuous	20 (71.4%)	7 (31.8%)	8.863*	**0.012**
Neo-angiogenesis	1 (3.6%)	5 (22.7%)		
Sub-capillary Plexus	9 (32.1%)	5 (22.7%)	4.352*	0.113
Nailfold capillaroscopy pattern				
Early	13 (46.4%)	1 (4.5%)	11.826*	**0.003**
Late	1 (3.6%)	4 (18.2%)		
Active	14 (50%)	17 (77.3%)		
Avascular score	1.18 ± 0.82	2 ± 0.62	–3.909•	**<0.0001**
Pulsatile index (PI)	0.81 ± 0.1	0.93 ± 0.11	–2.744•	0.009
Systolic/Diastolic ratio	2.66 ± 0.29	3.6 ± 0.49	–5.628•	**<0.0001**

DCSS: diffuse cutaneous systemic sclerosis; LCSS: limited cutaneous systemic sclerosis; SBP: systolic blood pressure; DBP: diastolic blood pressure; RVSP: right ventricular systolic pressure; ESR: erythrocyte sedimentation rate; GFR: glomerular filtration rate; AST: aspartate transaminase; ALT: alanine transaminase; LDL: low density lipoprotein; HDL: high density lipoprotein; HPF: high power field; RRI: renal resistive index; PI: pulsatile index.

High RRI is associated positively with age, period of disease and Raynaud’s phenomenon, DSS, and avascular score (p < 0.05), and adversely with creatinine clearance, eGFR, and capillary density (p < 0.05) (**Table 3**). The progression of the avascular NFC score was significantly positively linked to RRI and the S/D ratio (p < 0.05), indicating end-organ damage associated with nailfold avascularity. Additionally, eGFR correlated negatively with age, age at disease onset, proteinuria, uric acid level, serum creatinine level, RRI, pyuria, and S/D index (p < 0.05), and positively with creatinine clearance (p < 0.000).

**Table 3.** Correlation of renal resistive index with demographic, clinical, laboratory data, and nailfold capillary findings.

	**Resistive index (RRI)**	
	**R**	**P-value**
**Age**	0.323*	**0.023**
Age of disease onset	0.206	0.157
**Disease duration (years)**	0.320*	**0.025**
BMI (kg/m^2^)	–0.277	0.054
SBP (mmHg)	0.093	0.525
DBP (mmHg)	–0.043	0.769
**Duration of Raynaud's (years)**	0.286*	**0.049**
**Disease Severity Score**	0.284*	**0.048**
Total leukocytic count (10^3/μl)	–0.008	0.956
**Haemoglobin (mg/dL)**	–0.311*	**0.029**
Platelets (10^3/μl)	0.147	0.312
ESR (mm/hour)	0.021	0.883
AST (u/l)	–0.051	0.730
ALT (u/l)	0.006	0.969
Triglycerides (mg/dl)	–0.001	0.994
Cholesterol (mg/dl)	0.060	0.684
LDL (mg/dl)	–0.025	0.864
HDL (mg/dl)	0.022	0.882
Serum creatinine (mg/dl)	0.149	0.306
Urea (mg/dl)	0.078	0.595
Uric acid (mg/dl)	0.038	0.794
**Creatinine clearance (ml/min)**	–0.342*	**0.016**
**eGFR (ml/min/1.72m^2^**	–0.299*	**0.037**
Pus cells	0.162	0.267
Red cells	0.016	0.911
Protein/creatinine	0.237	0.101
**Capillary density/mm**	–0.317*	**0.026**
Mean capillary length (micro)	–0.165	0.257
Mean capillary width	–0.049	0.741
**Avascular score**	0.362*	**0.011**
**Pulsatile index (PI)**	0.384**	**0.006**
**Systolic/Diastolic ratio**	0.893**	**0.000**
Duration (years)	0.261	0.103

Univariate logistic regression analysis identified the following parameters as positive predictors of high RRI: age > 43 years, dcSSc, DSS > 5, protein/creatinine ratio > 0.21, capillary density ≤ 6, NFC pattern, and avascular score > 1 (p < 0.05). The multivariate analysis highlighted age > 43 years and late nailfold capillary patterns as the most significant factors connected with high RRI (odds ratio with 95% CI; **Table 4**).

**Table 4.** Univariate and multivariate regression analysis for predictors associated with high RR index.

	**Univariate**		**95% C.I.for OR**		**Multivariate**		**95% C.I.for OR**	
	**P-value**	**OR**	**Lower**	**Upper**	**P-value**	**OR**	**Lower**	**Upper**
Age (years) > 43 years	**0.002**	8.667	2.230	33.682	**0.005**	**11.032**	**2.045**	**59.501**
DCSS extension of skin tightness	**0.038**	3.667	1.072	12.547	-	-	-	-
Disease activity	**0.013**	3.295	1.286	8.442	-	-	-	-
Disease activity score > 3	**0.025**	4.500	1.211	16.719	-	-	-	-
Disease Severity score > 5	**0.001**	11.400	2.695	48.227	-	-	-	-
Protein/Creatinine ratio >0.21	**0.019**	7.429	1.384	39.866	-	-	-	-
Capillary density ≤ 6	**0.008**	18.200	2.143	154.559	-	-	-	-
Tortuous	0.943	0.969	0.406	2.314	-	-	-	-
Nail fold capillaroscopy pattern	**0.010**	2.785	1.271	6.102	**0.010**	**23.591**	**2.151**	**258.733**

**Figure 2.**
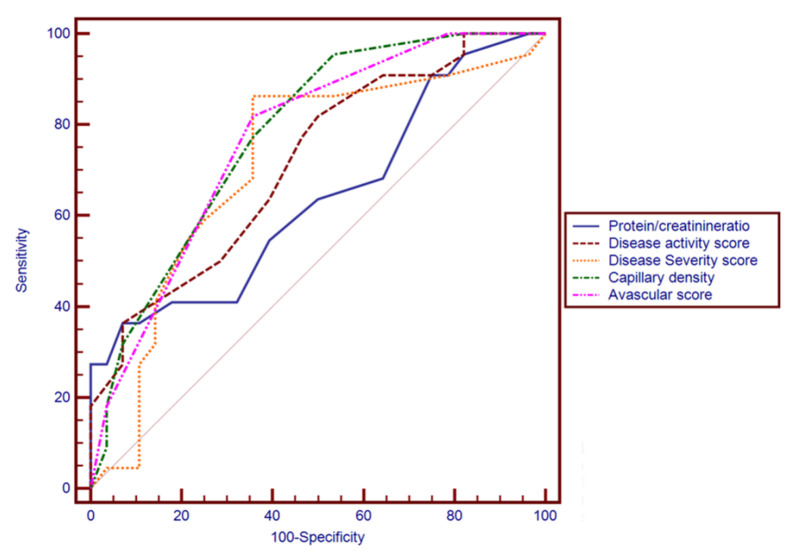
Receiver operating characteristic curve (ROC) for predictors of high renal resistive index among our systemic sclerosis patients.

The receiver operating characteristic (ROC) curve identified low capillary density (95.45%), S/D ratio (95.45%), and DSS > 5 (86.36%) as the most sensitive predictors of high RRI. Factors with high specificity included protein/creatinine ratio (92.9%), S/D ratio (88.89%), and PI (85.19%; **Figure 2**).

## DISCUSSION

Systemic sclerosis (SSc), known as scleroderma, is a chronic connective tissue disorder defined by endothelial cell malfunction and skin and internal organs fibrosis. Vascular dysfunction is a hallmark of SSc, affecting both the macrovasculature and microvasculature. ^22,23^ Clinically, renal manifestations occur in up to 50% of SSc patients,^24^ whereas histological proof of renal impairment has been detected in up to 80% of patients during autopsy studies.^25^

Like other autoimmune diseases, SSc predominantly affects females, with an F/M ratio of up to 7:1.^26^ Previous studies^6,7,27,28^ reported variable sex ratios (F/M: 4.7:1, 5.2:1, 6.9:1, 8.8: 1 respectively). Unexpectedly, current study had higher F/M ratio of 11.5:1.

Herein, the mean age of patients was 40.56 ± 10.8 years. SSc Patients of other studies^6,7,17^ had higher mean age (49±13.8, 54.9 ± 13.8 and 55±15 respectively). Among the current cohort, 60% of patients experienced lcSSc, and 34% had dcSSc. This was somewhat consistent with a previous study.^28^ However, others^7,27^ reported nearly equal proportions of both lcSSc and dcSSc.

Symptomatic renal involvement, including proteinuria (6%), elevated creatinine levels (2%), reduced creatinine clearance (24%), and low eGFR (2%) ± hypertension (10%), was observed in 24% of the patients. Isolated hypertension (HTN) may develop in SSc patients with initially normal creatinine levels, and over time, up to 60% of patients may present with HTN associated with abnormal renal function or proteinuria within two years.^8^ However, serum creatinine has been identified as an inadequate indicator of renal damage in SSc patients due to its dependency on muscle mass and its limited ability to reflect true creatinine clearance.^29^ Proteinuria, on the other hand, has significant prognostic value.^30^ Additionally, eGFR is a more reliable marker for evaluating scleroderma nephropathy.^31^ Established renal impairment, particularly mitigated GFR, is correlated with poorer outcomes in SSc.^5,31^

This highlights the importance of recognising subclinical nephropathy in SSc, a condition often underestimated despite its potential implications for disease progression and management.

Subclinical vascular renal haemodynamics can now be evaluated through non-invasive measurement of the RRI, derived from intrarenal Doppler arterial waveforms using the formula: (PSV – end-diastolic velocity) / PSV.^32^ Doppler indices from interlobar arteries have been established as accurate indicators of renal resistance and reliable predictors of adverse outcomes in chronic nephropathies.^33^ Studies have demonstrated that compared to healthy controls, SSc patients revealed normal creatinine levels in serum and no comorbidities exhibit elevated RRI and systolic/diastolic (S/D) ratio values.^11,34^

In the current study, the median RRI was 0.66 (0.61–0.71), the median PI was 0.86 (0.81–0.97), and the median S/D ratio was 3.0 (2.6–3.6). Other studies^11,27^ showed median RRI [0.69 (0.65–0.73), 0.6(0.45–0.77) respectively] and median S/D ratio [3.3 (2.8–3.7), 2.5 (1.8–5.4) respectively]. In this cohort, 44% of patients exhibited elevated RRI values.

Resistance indices are hypothesised to rise during the early stages of renal damage progression while the GFR remains normal or is only slightly diminished. This initial decline in mGFR is attributed more to a reduced intrarenal blood supply than to diminished filtration capacity. As such, RRI is an early indicator of renal damage and a valuable marker of asymptomatic renal involvement.^11^

Herein, based on RRI values, patients were allocated into two groups: Group 1 exhibiting normal RRI, and Group 2 experienced high RRI. This classification highlights the potential role of RRI as a tool for detecting early subclinical renal impairment in SSc patients.

A highly significant age difference was observed between the two groups, with patients in Group 2 being older (p < 0.000). This finding aligns with earlier research.^6,27^ Nevertheless, other studies^11,35^ found no significant age differences between the groups. Regarding disease duration, previous research^11,27^ did not reveal a significant difference between the groups, which was the case in the current study. Conversely, others^35,36^ have found longer disease durations in patients with high RRI.

Fibrosis of the skin and contractures have been proposed as clinical markers of scleroderma nephropathic vascular changes and internal organ damage progression.^6^ In the current study, the extent of skin fibrosis manifested a significant difference between the groups (p = 0.034), with Group 2 patients exhibiting a higher prevalence of the dcSSc type. Group 2 patients reported more dyspnoea (p = 0.003), although there was no significant difference in the presence of interstitial lung disease (ILD). Disease severity, as measured by the Medsger DSS, was significantly raised in Group 2 (p < 0.01), consistent with findings from Rosato et al. (2018)^7^ and Leodori et al. (2021).^27^

Moreover, no significant difference was detected between the two groups concerning serum creatinine levels, a finding that was aligned with the outcomes of other researchers.^6,11^ These findings suggest that asymptomatic renal vasculopathy with subclinical renal impairment commonly occurs in SSc and does not correlate with serum creatinine levels. Additionally, no significant differences between the groups in eGFR or creatinine clearance were detected. However, patients in Group 2 revealed significantly elevated proteinuria levels (p = 0.01), in contrast to the outcomes of Rosato et al. (2012),^11^ which found no significant difference in proteinuria.

NFC microvascular abnormalities have been linked to end-organ damage and severe vascular phenomena in SSc patients and may correlate with higher RRI values.^37^ In the current study, late and active nailfold capillary patterns and neoangiogenesis revealed a significant rise in Group 2 patients (p = 0.012). These outcomes corroborate the outcomes of others,^6,7^ who reported the progression of capillaroscopic patterns in patients exhibiting higher RRI values.

Significant positive correlations were observed between the RRI and several clinical parameters, including age, duration of disease, Raynaud’s phenomenon duration, DSS, and avascular score (p < 0.05), indicating a relationship between end-organ fibrosis, vasculopathy, and nailfold avascularity with marked capillary loss. These findings align with prior studies that revealed a positive linear association between RRI and factors such as age, DSS, Raynaud’s phenomenon duration, and advanced capillaroscopic patterns.^6,7,27^

In contrast, significant negative correlations of RRI were found with creatinine clearance, eGFR, and capillary density (p < 0.05). These results corroborate prior research,^7,17,27,28^ which showed negative correlations between renal hemodynamic parameters and estimated and measured GFR. It has been previously reported that mGFR significantly hinders capillaroscopic damage progression.^11^ The current study found that eGFR had negative correlations with several factors, including patients’ age, age of disease onset, serum creatinine levels, uric acid levels, pyuria, proteinuria, RRI, and the systolic/diastolic index (p < 0.05). Conversely, eGFR is significantly associated with creatinine clearance (p < 0.000). These findings contrast with studies that have suggested renal impairment is not linked to factors such as age, disease duration, organ damage, skin score, or antibody profile.^38^Leodori et al. (2021) concluded that patient age, capillaroscopic pattern, and disease activity index are risk factors for renal damage and predictors of mortality.^27^ Logistic regression analysis of the current data identified several positive predictors of high RRI, including age > 43 years, dcSSc, disease severity score > 5, protein/creatinine ratio > 0.21, capillary density ≤ 6, NFC pattern, and avascular score > 1 (p < 0.05). Multivariate regression analysis ascertained age > 43 years and late nailfold capillary pattern as the strongest predictors of high RRI. Similarly, Gigante et al. (2021) reported that age, disease duration, and disease activity score were positive predictors of high RRI in SSc patients.^28^

This work had some limitations, among which the relative small number of enrolled patients from a single tertiary centre over short period, the relative female predilection, pulmonary hypertension diagnosis relied only on clinical and echocardiographic data, lack of further prospective evaluation, correlation with RNA polymerase III and possible reversibility of renal resistive index in response to relatively stronger vasodilators. Thus, further multicentre studies with larger sample sizes and extended follow-up are recommended for a more comprehensive evaluation of renal vasculopathy and its associated risk factors.

In conclusion, asymptomatic renal vascular affection is common in SSc patients. The RRI can be considered an indicator of renal vasculopathy and a predictor of internal organ involvement and mortality. Doppler indices of intrarenal arterial stiffness provide a non-invasive diagnostic tool for assessing renal damage in SSc patients, even those with normal GFR, and are correlated with capillaroscopic microvascular data.

## Data Availability

All available upon request.
